# μSPIM Toolset: A software platform for selective plane illumination microscopy

**DOI:** 10.1016/j.jneumeth.2020.108952

**Published:** 2021-01-01

**Authors:** Daniel Saska, Paul Pichler, Chen Qian, Christopher L. Buckley, Leon Lagnado

**Affiliations:** Sussex Neuroscience, University of Sussex, Brighton BN1 9QG, UK

**Keywords:** selective light-sheet microscopy, spim, micromanager, acquisition, toolbox, toolset, uspim, μSPIM

## Abstract

**Background:**

Selective Plane Illumination Microscopy (SPIM) is a fluorescence imaging technique that allows volumetric imaging at high spatio-temporal resolution to monitor neural activity in live organisms such as larval zebrafish. A major challenge in the construction of a custom SPIM microscope using a scanned laser beam is the control and synchronization of the various hardware components.

**New Method:**

We present an open-source software, μSPIM Toolset, built around the widely adopted MicroManager platform, that provides control and acquisition functionality for a SPIM. A key advantage of μSPIM Toolset is a series of calibration procedures that optimize acquisition for a given set-up, making it relatively independent of the optical design of the microscope or the hardware used to build it.

**Results:**

μSPIM Toolset allows imaging of calcium activity throughout the brain of larval zebrafish at rates of 100 planes per second with single cell resolution.

**Comparison with Existing Methods:**

Several designs of SPIM have been published but are focused on imaging of developmental processes using a slower setup with a moving stage and therefore have limited use for functional imaging. In comparison, μSPIM Toolset uses a scanned beam to allow imaging at higher acquisition frequencies while minimizing disturbance of the sample.

**Conclusions:**

The μSPIM Toolset provides a flexible solution for the control of SPIM microscopes and demonstrated its utility for brain-wide imaging of neural activity in larval zebrafish.

## Highlights

• μSPIM Toolset is open-source software for Selective Plane Illumination Microscopy.
• μSPIM synchronizes two scanned laser beams with various hardware components.
• Built around MicroManager software to integrate with range of cameras and hardware.
• μSPIM calibration procedures make it independent of microscope optical design.
• μSPIM images 100 planes through the brain of larval zebrafish at 1 Hz.

## 1 Introduction

Selective Plane Illumination Microscopy (SPIM) is a powerful method for 4D imaging of biological samples at a high spatio-temporal resolution (Ahrens et al., 2013; Power and Huisken, 2017). This is achieved by excitation of fluorescent structural or functional reporters expressed in the sample by a few micron thick light sheet and simultaneous recording by an orthogonally positioned high-resolution camera focused on the plane of excitation. Rapid movement of the sheet through the tissue then allows for volumetric recordings with cellular resolution. While SPIM imaging has been originally used for the study of developmental processes in a number of animal models including embryos of C. *elegans* (Rieckher et al., 2015), Drosophila (Keller et al., 2010) and zebrafish (Kobitski et al., 2015; Wan et al., 2019), more recently it has also found its use in functional imaging as a complement to already established imaging methods such as two photon imaging. By providing the ability to image much larger volumes of tissues while maintaining temporal and spatial resolution, SPIM provides the ability to investigate interaction of much larger neuronal populations as illustrated by imaging of the whole nervous systems in Drosophila embryos and larvae (Chhetri et al., 2015; Lemon et al., 2015), C. *elegans* (Ardiel et al., 2017) and whole-brain imaging in zebrafish (Ahrens et al., 2012, 2013; Weisenburger and Vaziri, 2018).

While all implementations of SPIM share the common design including an illumination arm to create a 2D plane of illumination and an orthogonal collection arm that is forming the image onto the camera, some variations on this basic design have been developed with different applications in mind (Keller and Ahrens, 2015). These can be divided based on two main aspects of the design: firstly, the method of light sheet formation and secondly, the movement of the light sheet relative to the sample. The original approach creates a stationary light sheet using a cylindrical lens and then translates or rotates the sample with a moving stage. This implementation has been particularly useful in studies of development (Huisken and Stainier, 2009), but it is too slow to monitor the activity of neurons using, for instance, genetically-encoded calcium indicators. Applications in functional neuroscience therefore favour a configuration in which the sample is kept stationary while the light sheet is created using a fast scanning mirror that moves the light beam across a plane at least once per imaging frame, with a secondary mirror moving the beam in the z-dimension (Fig. 2B). This method has allowed "brain-wide" imaging of neural activity in live zebrafish with single neuron resolution and acquisition frequencies of ∼1 Hz (Ahrens et al., 2012, 2013; Keller and Ahrens, 2015).

Several SPIM solutions have been published but probably the most accessible in terms of both hardware and software are the OpenSPIM (Pitrone et al., 2013) and Open SPIM microscopy (Gualda et al., 2013) projects. Both these implementations are focused on imaging of developmental processes using the slower "moving sample" configuration and therefore have limited use if the aim is to image neural activity through volumes of the brain. The "moving light-sheet" configuration is more complex to control because it requires synchronization of several hardware components with millisecond temporal accuracy. For instance, movements of the imaging objective in the z-dimension must be synchronized with movement of the light-sheet to stay focused on the plane of illumination, often involving 50–100 planes per second. Perhaps for this reason, open software tools for the control of SPIMs in functional imaging experiments are not easily available and published research utilizing functional imaging generally does not include openly published and documented microscope control solutions. We aim to fill this gap by providing a control software solution, μSPIM Toolset, which adopts an open software approach for control of a SPIM microscope in which the light-sheet is scanned through a stationary sample. We achieve this by building μSPIM Toolset around Micro-Manager (Edelstein et al., 2010), an open-source platform widely used for control of microscopes, to provide a comprehensive user interface (Fig. 3) with a smooth learning curve. μSPIM Toolset has been designed to be used to control a range of custom-built microscopes, for which it is calibrated using semi-automated procedures. We demonstrate the utility of μSPIM Toolset for whole-brain imaging in larval zebrafish.

## 2 Design requirements

**Fig. 1:**
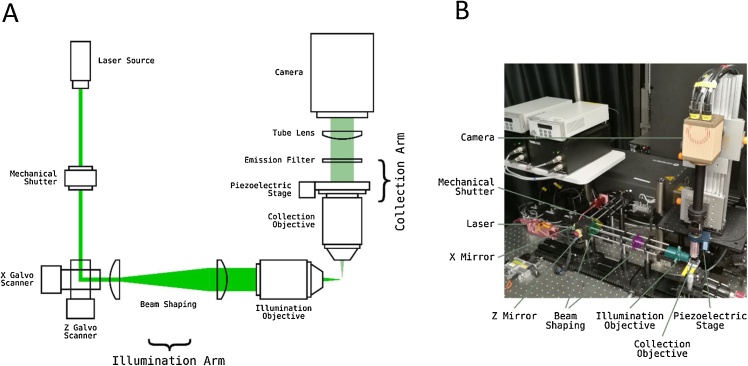
Light-Sheet Microscope Implementation. **(A)** Diagram outlining light-sheet microscope with two mirror galvanometers and a stationary sample. The light sheet is created from a laser beam by the’ X Galvo mirror’. The’ Z Galvo mirror’ then moves the sheet through the sample to create volumetric excitation. This is synchronized with a Piezoelectric stage that moves the imaging objective so that the excitation plane always coincides with the imaging plane. **(B)** A photo of an example light-sheet microscope setup with one light-sheet path. The main components are colour coded.

We start by listing the basic objectives of software controlling a SPIM microscope in relation to the microscope we have constructed (Fig. 1, see Methods). **1.** The illumination arm requires the control of the laser light-source, using either an internal and/or external shutter. Dual-colour imaging requires two independent laser sources to be combined. Depending on the laser model, power may be controlled on long time-scales using USB or RS232 interfaces and modulated on short time-scales by analogue signals, while shuttering requires digital signals. **2.** The beam has to be scanned in the x and z dimensions using fast galvanometer mirrors driven through analogue inputs. **3.** The collection arm must follow the beam and collect images that are in focus at different z-positions, which is achieved by using a piezo-electric mount for the objective, again controlled through an analogue signal. Finally, **4.** The camera acquisition must be accurately timed to collect frames at each z position, which in turn requires accurate calibration of the z mirror so that the z position of the light-sheet is set from the command signal. The software should then allow control of several features of the camera, including frame integration time, pixel binning and gain. Furthermore, it should support saving and retrieving images and movies, zooming, defining ROIs, quick and easy changes in imaging parameters and the ability to define various imaging protocols using different sequences of laser illumination

**Fig. 2:**
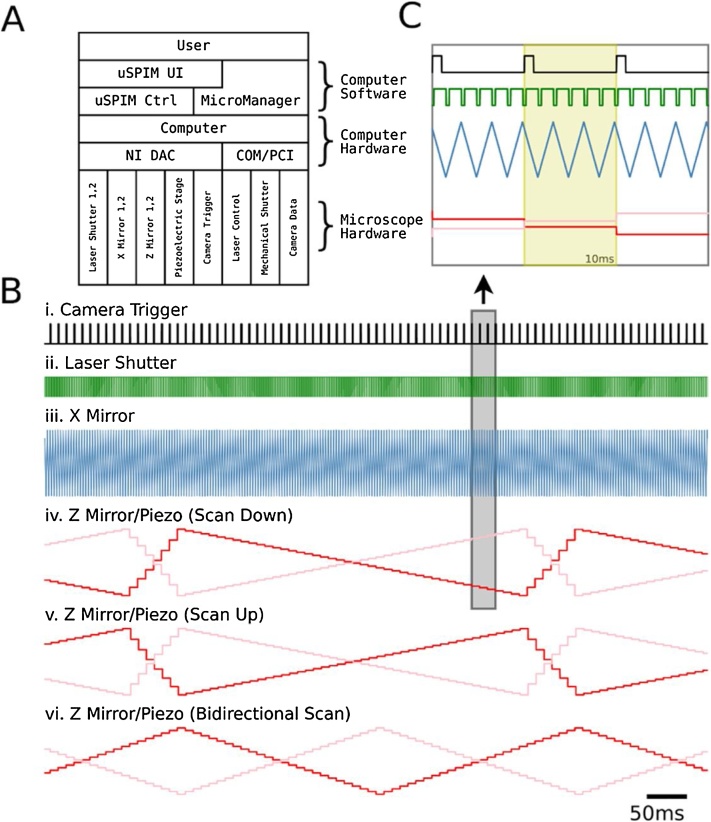
Light-Sheet Microscope Hardware Control: (A) Interaction of different components of the μSPIM Toolset-based setup in a typical acquisition setup. μSPIM Toolset provides control and synchronization of hardware through NI DAC with MicroManager controlling the camera and mechanical shutters through PCI and COM ports. **(B)** Traces of the command signals for a volume acquisition with 43 planes showing camera trigger (i.), laser shutter (ii.), X mirror signal (iii.), Z mirror (red) and Piezo (pink) signals for Scan Down (iv.), Scan Up (v.) and Bidirectional (vi.) acquisition modes generate by the μSPIM control software. **(C)** Magnification of boxed region in B with yellow region showing a single plane signal. Laser shutter signal is a result of a recording with Edge Masks enabled.

We built μSPIM Toolset around the open-source software Micro-Manager (Edelstein et al., 2010), which is based on ImageJ (Schindelin et al., 2015), because it immediately offers the ability to integrate different hardware from a wide range of manufacturers through specific plugins, including a range of cameras, light-sources and shutters required for a SPIM. It provides an integrated environment for image acquisition and a very wide range of post-acquisition processing capabilities through ImageJ. Micro-Manager does not, however, provide hardware triggering, analogue control and monitoring with the precision required for volumetric imaging of neural activity. For this purpose, we used a National Instruments DAC card (NI PCIe-6738) and wrote an executable ‘μ*SPIM Control*’ to allow interaction between Micromanager and hardware through its own user interface. μSPIM Control synchronizes internal laser shutters, x and z mirrors, the objective piezo stage and all camera triggering, leaving laser power, external mechanical shutters and camera operating properties under control of Micro-Manager (Fig. 2A). μSPIM Toolset therefore provides both accessibility and versatility, retaining the user’s ability to select hardware best suited for the particular setting, as long as it is supported through a Micro-Manager plug-in (The current range can be viewed at https://micro-manager.org/wiki/Device%20Support).

## 3 Principles of operation

μSPIM Toolset consists of two main components: a Java plugin for MicroManager which facilitates all user interaction and configuration, and a C++ control executable which interacts with the National Instruments board to produce hardware control signals (Fig. 2). These support the control of two scanning X mirrors for light sheet formation, two scanning Z mirrors and one piezoelectric stage for Z motion of the collection objective, two laser shutters for laser masking and one trigger for the camera acquisition control and synchronization with other hardware. After an appropriate setup, the Java plugin facilitates all necessary tools for calibration and control of the hardware, providing the users with an intuitive interface without requiring the need for specialized computer knowledge or programming, while allowing flexibility provided by MicroManager. Fig. 2 illustrates the control diagram, outlining the interaction between the user and the light-sheet microscope hardware (Fig. 2A) as well as the temporal sequence of the control signals (Fig. 2B and C).

**Fig. 3:**
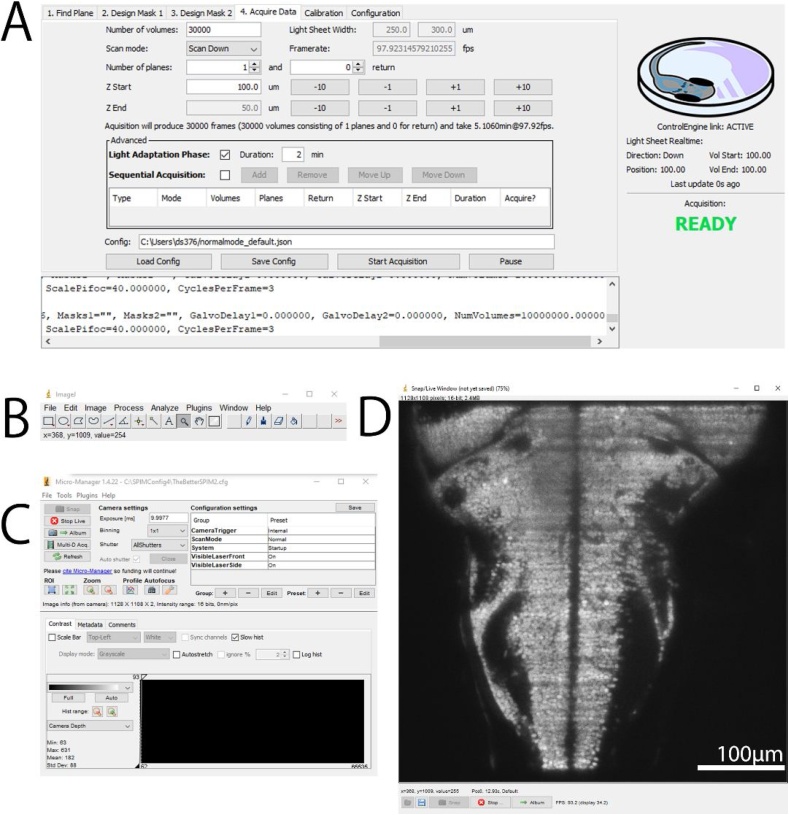
μSPIM Toolset User Interface. Following a common MicroManager design, the control interface is separated into several components: μSPIM Toolset-provided plugin window with control over the light-sheet generation including calibration and acquisition routines shown in A, basic ImageJ tools shown in B and MicroManager interface providing control over the acquisition hardware shown in C and live view of the camera shown in D. The shown user interface has been captured during the acquisition from a 7 dpf larval zebrafish from the *Tg(elavl3:H2B-GCaMP6f)* transgenic line.

A typical user interface for data acquisition includes the μSPIM Control window (Fig. 3A) and the standard MicroManager windows (Fig. 3B-D). The primary Micromanager window (Fig. 3C) provides access to all functionality related to the acquisition, such as live mode, binning, ROI selection or exposure time. The μSPIM plugin (Fig. 3A) provides access to the variables used for light-sheet formation, such as light-sheet width, volume depth or number of planes as well as providing a set of calibration procedures described in the Calibration section below. During scan mode, the image can be viewed in the classical MicroManger *live* window (Fig. 3D) which also allows the acquisition of snapshots. While acquisition can be manually started through the MicroManager interface, the μSPIM plugin interface provides an acquisition routine executing a synchronized start of acquisition and illumination, making the process of acquiring data (namely volumes) simpler and more robust.

**Fig. 4:**
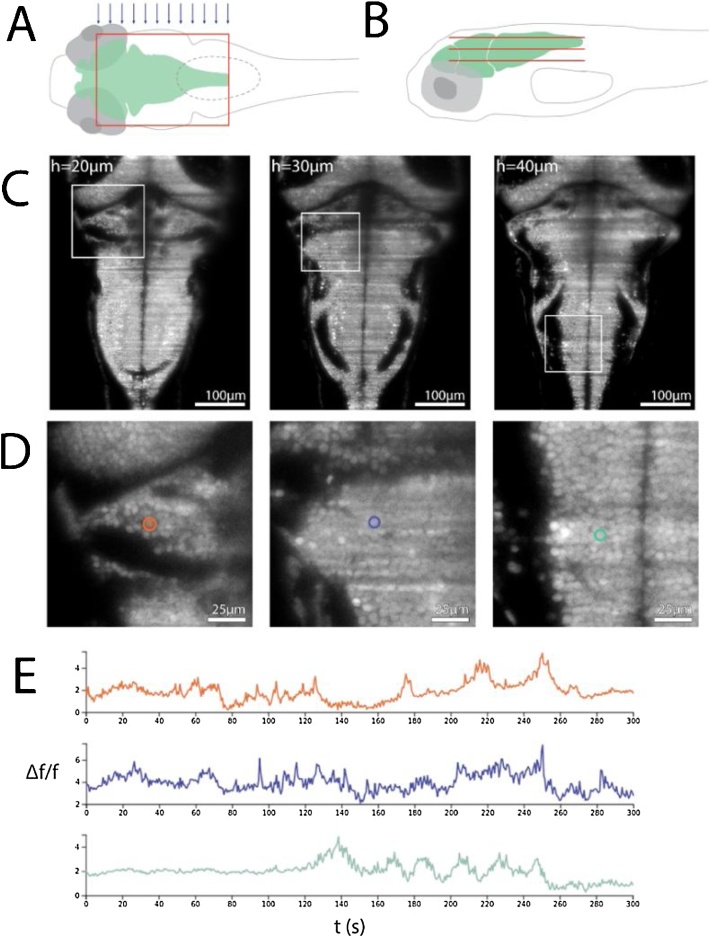
Imaging larval zebrafish: (A and B). Schematic of a single light sheet covering the hindbrain of a larval zebrafish from above (A) as well as a number of light sheets (constituting a volume) from the side (B, sheet size and spacing are not to scale). **(C)** Transgenic zebrafish expressing the calcium reporter GCaMP6f in the cell nuclei of all neurons (*Tg(elavl3:H2B-GCaMP6f)*) were embedded in agarose and positioned so that the laser entered the brain from the side (blue arrows in A). Representative sections of the volume taken at 20, 30 and 40 μm depths at a frequency of 1.6 volumes/sec and an integration time of 10.2 ms per section. The step size was 2 μm, hence representative sections are 5 sections apart. The laser was set to 1.8 mW. **(D)** magnified view of boxed areas in C. Single cell nuclei are clearly visible and three examples are highlighted. **(E)** Activity of single cells highlighted in D.

Taking full advantage of the stationary sample and fast acquisition speeds, we used μSPIM Toolset to monitor the activity of neurons across most of the brain of larval zebrafish expressing the nuclear localised Ca^2+^ reporter GCaMP6f panneuronally (*Tg(elavl3:H2B-GCaMP6f*), Fig. 4). Larvae were embedded so that the laser excited the sample from the side (arrows in Fig. 4A). The width of the sheet spanned 500 μm and covered the entire hindbrain, the cerebellum and parts of the optic tectum. Single planes were recorded at a frequency of 98 Hz. The volume was set to cover a distance of z = 100 μm and consisted of 50 planes with a spacing of 2 μm. The piezo returned to its original position within 10 frames yielding an overall imaging frequency of 1.6 volumes per second. Fig. 4C shows three representative sections at depths of 20, 30 and 40 μm, respectively. The lateral and axial resolution of the microscope were 0.7 ± 0.1 μm and 5.4 ± 0.5 μm, respectively (mean ± sd, full width at half maximum, see Methods) and the nuclei of individual cells are clearly visible in the cerebellum, the anterior and the posterior hindbrain, respectively (Fig. 4D) and their activity over time is shown in Fig. 4E.

## 4 Computing requirements

μSPIM Toolset relies only on minimal third-party software for its operation. This includes MicroManager for integration of acquisition hardware (laser control, light path shutter and camera) and National Instruments drivers, in order to interface with the National instruments DAC card. Naturally, drivers and accompanying software must be installed for the selected hardware (such as lasers or camera) in order to ensure MicroManager can communicate with those parts properly. Due to the continuous high data throughput, it is recommended to use a secondary computer for stimulation and/or other intensive tasks to avoid potential performance issues caused by the multiple tasks competing for computational resources or ensuring that this is not the case when a single machine is used.

The computer specifications necessary for optimal function and acquisition of the setup are highly dependent on the acquisition requirements. For large field of view and high acquisition speeds, the acquisition produces large datasets, making the process highly storage-dependent. State-of-the-art acquisition cameras are able to reach 4 megapixel resolution at 100 Hz and 16 bit depth, producing a theoretical throughput of 838.9 MB/s. This poses a write speed requirement higher than that which can be satisfied by a regular SATA3 storage interface which is limited to a theoretical maximum of 600 MB/s. It is therefore highly recommended to utilize a faster storage solution, such as fast PCI-based solid state disk (SSD) storage (e.g. Intel Optane 905p) or hardware SSD RAID. The acquisition is not highly CPU dependent and neither μSPIM Toolset nor MicroManager take a significant advantage in parallel processing. A state-of-the-art consumer line 4- or 6-core CPU is sufficient for optimal performance.

μSPIM Toolset supports control of two independently calibrated laser paths. Each path consists of a visible light laser, two scanning mirrors and one laser path shutter (Fig. 1). The laser path shutter must be supported by MicroManager and is used to block light between periods of acquisition and thus reduce photo-bleaching of the sample. One of the two scanning mirrors is used for light sheet formation and the second is used for Z movement. The laser should provide digital or analogue shuttering capability. All other devices are controlled using an analogue signal produced by the National Instrument DAC adapter and should support inputs in appropriate voltage ranges (commonly ±10 V). μSPIM Toolset allows for incomplete configurations e.g. single light path with unmodulated laser and single sheet-generating scanning mirror would provide minimal functionality, however most of the features of the μSPIM Toolset would be unavailable. To support the full functionality, the National Instruments DAC card must support at least 8 analogue outputs with no need for analogue or digital inputs as the control is fully feed-forward and does not require any feedback from the hardware.

## 5 Calibration

**Fig. 5:**
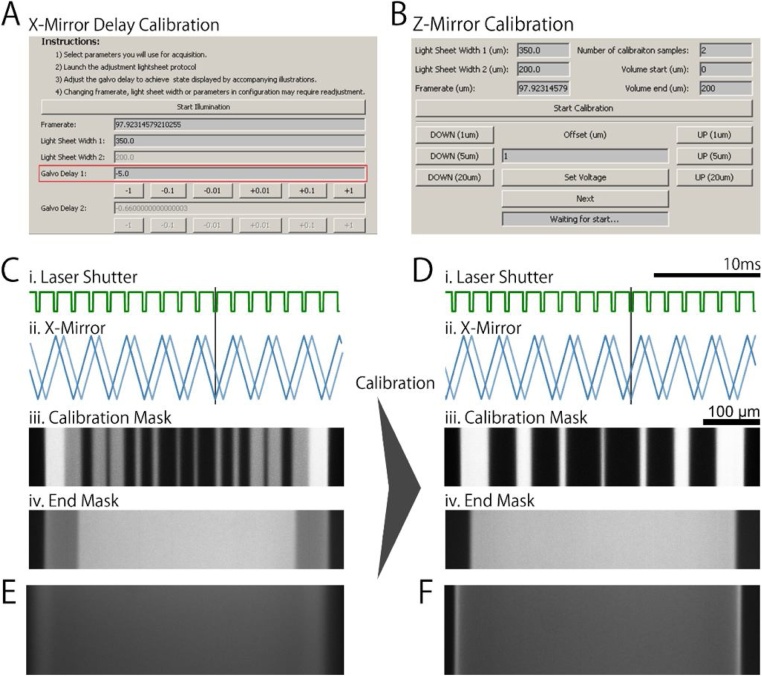
μSPIM Toolset Calibration μSPIM Toolset provides the user with calibration tools which can be used to assess and correct the performance of the individual microscope elements. **(A)** and **(B)** show the calibration interface for X mirror with respect to the laser shutter when using masks and for Z mirror, respectively. **(C)** The movement of the X mirror galvanometer lags behind the supplied signal, resulting in artefacts when using laser masks. **(i.)** shows the laser shutter signal (green) and **(ii.)** shows the X mirror signal (blue) and actual mirror movement (light blue). Black bar highlights the misalignment between the shutter signal and mirror movement when the X mirror signal is not calibrated and no delay is introduced. **(iii.)** shows a mask used for calibration, emphasizing the difference between uncalibrated and calibrated system. **(iv.)** shows the corresponding light sheet produced with poor calibration for the mask signal in (i.). **(D)** is analogous to **(C)**, showing a well calibrated system where the laser shutter **(i.)** is synchronized X mirror movement **(ii.)**, producing overlapping masks shown in **(iii.)** and **(iv). (E)** Uncalibrated Z-Mirror results in loss of focus through the volume. **(F)** After calibration, the light sheet is always in focus throughout the volume scan. All figures illustrating the light sheet in both uncalibrated and calibrated scenarios were acquired by scanning the laser through a fluoroscein solution.

The spatial scales of the signals are greatly dependent on the chosen hardware and thus require the user to perform a set of calibration procedures to ensure appropriate alignment of the hardware prior to the first use of the software for acquisition. Subsequently, the calibration needs to be done only when hardware components are changed and routinely when it is suspected the software has gone out of calibration. Here and in Fig. 5 we outline the principles of the initial calibration of the different components sufficient to achieve an acquisition-ready configuration.

### 5.1 X-Mirror

Proper X-plane mirror calibration is essential for the correct assessment of size of the illuminated region when starting acquisition. To calibrate the X mirror, it is necessary to know the size of the field of view (or pixel size). This can be calculated as the effective camera image sensor size divided by the magnification of the collection objective or by measuring a standard stage calibration slide under the microscope. Using this measurement, the X Mirror scale can be adjusted such that a sheet of a particular width matches the corresponding number of the pixel. In the case of two illumination paths, this procedure should be repeated for both paths separately. This calibration should be carried out such that the x-mirror moves around its central position when energized (i.e. with zero volts driving signal) and the illumination arm should be adjusted such that this central position is centred on the field of view.

### 5.2 Piezoelectric stage

Piezoelectric stages are generally factory-calibrated to move a predefined distance by set voltage difference (e.g. 1 μm per 40 mV for the PIFOC model) to which the Piezo Scale setting should be set. The unit is mV/μm and for the previous example this setting would be set to 40.0. μSPIM Toolset also provides a Piezo Offset setting which can be used to adjust voltage offset in software. For optimal operation, it is suggested to adjust the offset such that a plane at 0.0 μm is near the top of the available range. Setting the offset could be beneficial, for instance, when imaging at multiple depths in a sample so that when 0.0 μm is positioned at the top of the sample it serves as a reference while allowing the full range of the piezoelectric stage to be utilized.

### 5.3 Z- mirror

Next, it is necessary to calibrate the relative movement of the Z-plane mirror(s) with respect to the movement of the now-calibrated Piezoelectric stage. This can be done using a calibration procedure provided by μSPIM Toolset which steps the stage through a user-defined number of levels across a volume of choice and allows the user to adjusts the Z mirror voltage for each level such that the laser sheet is in focus. Finally, a Z Mirror scale is generated which can then be used to adjust settings.

### 5.4 X-mirror lag compensation

The X Mirror movement lags the signal which can be problematic when using laser masks (discussed in Section 6.1). To correct for this, μSPIM Toolset provides a simple calibration process during which a distinct mask is displayed and the mirror lag is adjusted by the user until the masks perfectly overlap (Fig. 5).

## 6 Data acquisition using μSPIM Toolset

For the acquisition of a single plane, the X mirror galvanometer has to be scanned at least once across the full range of the desired light sheet width and back to its original position (Fig. 2, showing 3 scans per frame). To ensure uniform illumination between frames, μSPIM Toolset takes control over the camera using a rising edge trigger signal synchronized with the X mirror (Fig. 2). Generation of a volume recording is provided by a synchronous movement of the Z Mirror and the Piezoelectric Stage between frames, acquiring consecutive frames from different heights in the samples (Fig. 2).

μSPIM Toolset implements three volume acquisition modes: Scan Down, Scan Up and Bidirectional mode. In the Scan Down mode, the volume frames are acquired starting from the top, moving down through the volume and then finally returning to the top of the volume to prepare for the acquisition of the next volume. Since the stage moves a relatively heavy objective, instant movement from the bottom to the top of the volume is not possible and fast movement can introduce oscillations in the stage’s position, reducing the quality of the recording. To eliminate this issue, a flyback period is introduced at the end of volume acquisition (shown in Fig. 2B v.), slowing down the return of the stage at the expense of several frames. The Scan Up mode offers similar functionality to that of Scan Down mode, however in the opposite direction. Bidirectional mode eliminates the need for the flyback period by alternating between Scan Down and Scan Up modes on consecutive volume acquisitions (Fig. 2B vi.) at the expense of unequal time period between frame acquisitions.

To allow for better distribution of sample illumination or illumination of occluded regions, μSPIM Toolset also supports the control of two separate paths, allowing for two light sheets of different widths.

### 6.1 Advanced acquisition methods

While basic acquisition described above may be sufficient for some applications, μSPIM Toolset is primarily intended for functional imaging with visible light which can disturb the live sample. To minimize the impact of the visible light, the μSPIM Toolset implements several procedures which can be used in applications where this is an issue.

**Fig. 6:**
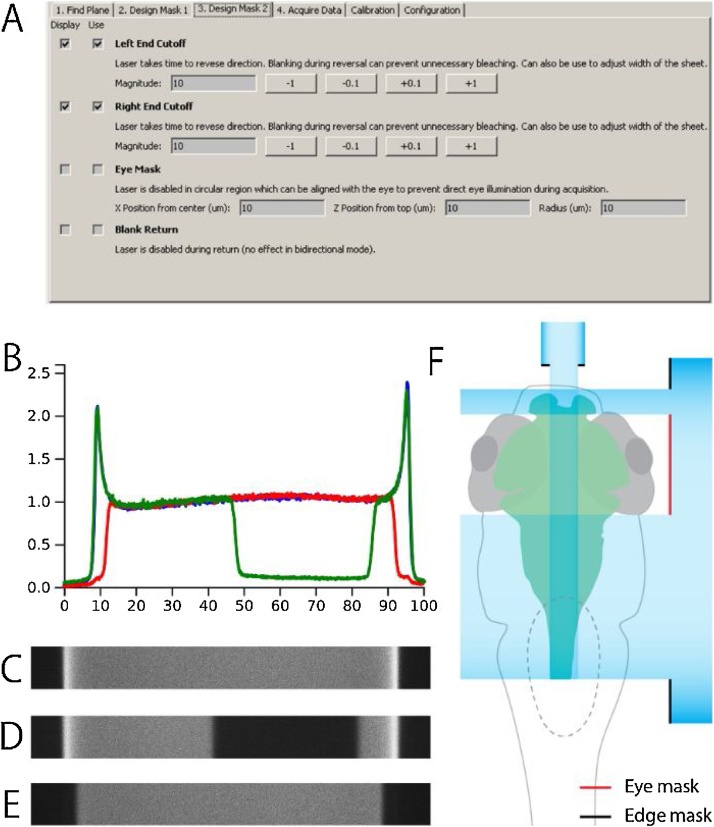
Light-Sheet Laser Masks. **A**. User Interface used to define laser masks allowing the user to display and use a number of masks for recordings: Edge masks help eliminate excess light during laser return as visible in D (with Edge masks enabled) compared to C (without any masks), Eye Mask allowing to turn the laser off in a specific region (such as eye of larval zebrafish) and Blank Return which turns the laser off during return (flyback), avoiding unnecessary photobleaching of the sample. **B** The median-normalized luminance intensity profiles of a light sheet in Fluorescein with no mask applied (blue, shown in C), edge masks (red, shown in D) and eye mask (green, shown in E). **F.** An illustration of how the ‘edge’ and ‘eye’ masks could be used in the context of imaging the brain of a larval zebrafish while minimizing illumination of the retina.

*1) Laser Masks:* Removing parts of the light sheet can be beneficial in some acquisition settings. μSPIM Toolset utilizes fast software shuttering to provide two main masks which may be beneficial for *in vivo* imaging: *Edge Masks*, removing the ends of the light sheet and thus reducing the regions which are over-illuminated due to slow reversion of the direction of the X mirror (Fig. 6) and *Eye Mask* which can be used to remove illumination from a selected region in the light sheet (Fig. 6), allowing for acquisition where a specific region must not be illuminated, such as the eye (Fig. 6F). Both masks are easily adjusted (Fig. 6A) and provide further flexibility in designing illumination sheets.

*2) Light Adaptation:* Zebrafish larvae have been shown to react to sudden light changes (Burgess and Granato, 2007), interfering with behaviours of interest. This proves problematic in visible light recordings as the light is commonly turned off between recordings to prevent photodamage to the sample, resulting in a sudden light change during the start of the recording. We provide a simple light adaptation functionality which places a light sheet in a position near the top of the sample prior to and in between recordings, where photodamage is of less concern and then moves the sheet into the defined position when the recording starts.

*3) Multi-recording sequence:* Similarly, to the adaptation period, sudden light changes can be reduced by consecutive acquisition of a number of recordings without the need for user input in between the recordings. This eliminates the concern of photodamage during non-acquisition time periods, allowing the laser to be turned on for the whole sequence of the recordings.

### 6.2 Data output format

**Fig. 7:**
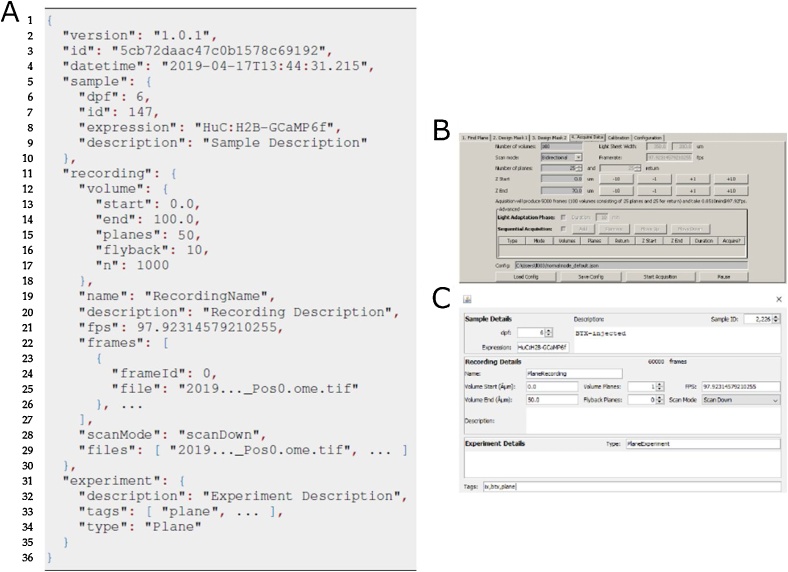
Acquisition using μSPIM Toolset & Output Data Format. μSPIM Toolset generates a separate metadata file (shown in **A**), supplementing metadata supplied by the MicroManager platform. The generated data contains information detailing the settings of the acquisition (such as the height of the recorded plane or number of planes in the recorded volume) based on the parameters set by the user in **B**. This information can be supplemented by the user to contain information about the sample imaged, comments about the experiment protocol as well as the recording itself to aid cataloguing of the data.

The image data output follows the MicroManager format with acquired image sequences being split into 4GB files accompanied by a metadata file (Metadata format can be found in the official MicroManager documentation: https://micro-manager.org/wiki/Files_and_Metadata). To accommodate for the extra information regarding the light sheet acquisition, μSPIM Toolset provides a separate metadata file which carries the information about the volume acquired as well as information about the experiment and sample provided by the user (Fig. 7).

## 7 Discussion

We have presented the μSPIM Toolset which provides a flexible software solution for the control of SPIM microscopes and demonstrated its utility for brain-wide imaging of neural activity in larval zebrafish (Ahrens et al., 2012). In contrast to other open source control solutions for light sheet microscopes, such as OpenSPIM (Pitrone et al., 2013) and Open SPIM microscopy (Gualda et al., 2013), μSPIM Toolset focuses on a microscope implementation better suited for functional imaging (as opposed to developmental imaging), filling a gap in available software solutions rather than competing with the existing implementations. With a range of in-built calibration protocols, μSPIM Toolset allows the user to select hardware based on the needs of the given application. The MicroManager platform on which μSPIM Toolset is built provides support for a wide range of acquisition hardware which will provide users with a range of options in customizing their instrument. Comprehensive documentation and the open source nature of the toolbox allows the user to adapt the software to advanced application and non-standard use-cases, overcoming the current limitations of the software.

While our framework offers great flexibility in the choice of hardware used, and is modular allowing incomplete implementations, μSPIM Toolset fundamentally depends on the National Instruments DAC and other digital-analogue converters are currently not supported. Adding support for other DAC devices is supported by the structure of the source code of our control software, but the modifications necessary may be quite extensive.

As previously outlined, the focus of μSPIM Toolset is primarily on the hardware flexibility aimed at a particular application (functional light sheet imaging) and thus some more complex functionality may not be supported out of the box. One notable example would be use of multiple lasers with various wavelengths which is required when imaging with multiple fluorescent reporters or markers: μSPIM Toolset is currently limited to providing support for two independent lasers. We have not yet explored the possibility of replacing one or either of these by a multi-colour light engine, but envisage that this will be possible given that micromanager supports a number of such devices. Again, advanced users may choose to modify our solution to support this functionality if required.

Being built around MicroManager, μSPIM Toolset inherits all of its limitations. For instance, while the list of acquisition hardware supported by the MicroManager platform is extensive, it is possible that some less common devices are not supported by the platform and thus cannot be used with our toolbox. Another notable limitation is the lack of support for the “rolling shutter” mode of acquisition in which the exposure of each line of pixels on the sCMOS sensor is delayed to coincide with the time at which those pixels are parfocal with the laser beam passing through the sample (also called electronic confocal slit detection (Hu et al., 2017)). Because only that single line of pixels is activated, much background fluorescence caused by scattered illumination of neighbouring areas is rejected, thereby improving resolution. We did not implement this mode of acquisition as a standard feature of μSPIM because it results in lower frame rates compared to the usual “global shutter” mode of acquisition in which all pixels are activated simultaneously.

While our framework does not support some use cases in its current implementation, it provides a comprehensive solution to the control of light sheet microscope with support for a wide range of both control and acquisition hardware while retaining a gentle learning curve supported by the well documented calibration and acquisition protocols. For this reason, we hope the μSPIM Toolset will be adopted in both standard and custom light sheet imaging use cases thanks to well-documented open source code base. Both documentation and source code is available for free from the μSPIM Toolset git repository (https://uspim.org).

## 8 Materials and methods

### 8.1 Optical design of SPIM used in this work

A partial parts list for the SPIM we constructed is provided in Table 1. The illumination arm (Fig. 1) was fed by one or two lasers through optical fibres (kineflex). The beam was reflected by a pair of X and Z Galvo scanning mirrors with 1 kHz bandwidth at deflection angles ± 0.2°. The beam was then expanded by a factor of 2.5 using a pair of achromatic lenses (f = 50 mm and 125 mm). The optimum beam parameters were calculated using calctool (http://www.calctool.org/CALC/phys/optics/f_NA). The illumination lens was f = 40 mm achromat. The whole of the illumination arm, beginning with the combining mirror, was mounted on an optical rail which was itself mounted on two translation stages (Thorlabs XR25C/M), one of which was slaved to the other. This arrangement allowed centering of the illumination beam on the field of view when the x-mirror was set to its position of zero offset, which is especially important for calibration of x-mirror displacement described above. With the rapid scanning of the X Galvo mirror, the laser line will form an illumination plane over the integration time of a single frame. The thickness of the beam waist was ∼7 μm, with a Rayleigh length of ∼440 μm. We therefore achieved a relatively uniform plane of illumination across half of the total field of view, which was 810 μm wide. The specimen stage was custom-designed according to application and manufactured using a 3D printer. The stage was attached to an x-y-z translation assembly (Thorlabs PT3) for positioning of the specimen.

**Table 1:** Parts list for construction of SPIM.

Mechanical Parts	Component (Manufacturer)
Optical table	1.4 × 1.8 × 0.2 m clean top on Micro-g Pneumatic and Rigid Legs (TMC Vibration Control)
Rail systems	Precision 100 mm Dovetail Optical Rails with PRC carriers (Newport)
Translation stages	XR25C/M (Thorlabs)
**Illumination arm**	
Lasers	LuxX+488 nm 60 mw (Omicron) Jive 561 nm 100 mW (Cobolt)
Optical Fibre and laser launch	Kineflex Fibre system (0.7 mm), 400−640 nm, 2 m, FC/APC connectors (Qioptiq)
Mechanical Shutter	SH05/M shutter and SC10 controller (Thorlabs)
Beam expander 2.5x	f = 50 mm achromat (AC254-050-A, Thorlabs) and f = 125 mm achromat (Edmund Optic)
Dual-axis galvanometer scanner	GVS102 - 2D Galvo System (ThorLabs)
Illumination lens	f = 40 mm achromat (#49-354-INK, Edmund Optics)
**Collection arm**	
Camera	ORCA-flash4.0 v2
Laser cleanup notch filters	488/10, and 561/10 (Chroma)
Detection objective	16X/0.8NA CFI LWD Plan Fluorite (Nikon)
Piezo	PIFOC E-665 amplifier/controller + P-725 (400 um travel range from Physike Instrumente)
Emission filter	535/35 (xf3007, Omega)
Tube lens	f = 200 mm achromat (AC508-200, Thorlabs)
Mounting of collection arm	X-95 mounting system and carriers (Linos)
**Computing**	
Custom built PC incorporating Intel® Xeon® processor E5-2600	
Frame Grabber	Firebird Camera Link (2xCLM-2PE8) (Active Silicon)
DAC card	PCIe-6738 (National Instruments)

For detection, a 16X/0.8NA Nikon CFI LWD Plan Fluorite objective was placed perpendicularly to the illumination plane to collect the emitted fluorescence signal. The excitation light was rejected by the emission filter and then a tube lens of 200 mm focal length (AC508-200-A-ML, Thorlabs, Inc) used to project an image onto the sensor of the Hamamatsu Flash4.0 sCMOS camera (13 × 13 mm CCD, so that each pixel imaged an area of ∼0.16 μm2 using a 16x objective). The objective lens was mounted on a piezoelectric stage (P-721 PIFOC High-Precision Objective Scanner, Physik Instrumente Ltd) and its movement synchronized with the Z Galvo scanner to make sure the illumination plane is always in the imaging focal plane. The objective and PIFOC scanner were themselves mounted on a manual stage for finding the focal plane before starting acquisition.

### 8.2 Characterising lateral and axial resolution of the microscope

To characterise the lateral (x/y) and axial (z) resolution of our SPIM we used fluorescent sub-diffraction size beads (0.1 μm diameter “fluospheres”, Invitrogen), suspended in 1 % low melting point agarose (Sigma-Aldrich). Using a 200 μm wide light sheet and a step-size of 0.5 μm several volumes of the suspended beads were acquired. 22 non-overlapping beads were used to determine lateral and axial resolution. Axial resolution was determined by fitting a gaussian function to the z-profile running through the point of highest intensity (the centre of the bead). The position of the peak of that function was used to determine the imaging plane running through the centre of the bead and a 2D gaussian where sigma(X) = sigma(Y) was fit to that plane. Using this procedure, we determined a lateral resolution of 0.7 ± 0.1 μm (mean ± sd, full width at half maximum, n = 22 beads) and an axial resolution of 5.4 ± 0.5 μm.

### 8.3 Imaging brains of larval zebrafish

All procedures were in accordance with the UK Animal Act 1986 and were approved by the Home Office and the University of Sussex Ethical Review Committee. Transgenic zebrafish larvae of undetermined sex expressing the nuclear localised H2B-GCaMP6f calcium reporter panneuronally (*Tg(elavl3:H2B-GCaMP6f*) (Chen et al., 2013; Dunn et al., 2016)), were imaged at 6–7 days-post-fertilisation (dpf) as a part of an ongoing investigation into Optomotor Response in larval zebrafish. All lines were maintained in the nacre background to limit pigmentation. Larvae were paralysed by positioning them sideways in a small slit of PDMS (Sylgard184, Dow Crowning) on a coverslip (Pichler and Lagnado, 2019) and injecting 0.25 mM a-Bungarotoxin (Tocris Bioscience) into their heart. They were then embedded dorsal side up in 2 % low-melting-point agarose (Biogene) in E2 medium (Brand et al., 2002) a 22 mm x 22 mm coverslip and placed into a custom glass-walled 3D printed chamber. After filling the chamber with E2 medium, the majority of the agarose that fell between the laser source and the larva’s head was removed with a scalpel to maintain sufficient stability of the sample while minimizing the way the laser travelling through the agarose. The chamber was positioned underneath the objective so that upon turning on the laser, it would not hit the head, and the eyes in particular, but rather the tail of the fish. Once the imaging and excitation planes were successfully aligned, the larva’s brain and in particular the desired brain region was approached. Finally, experimental parameters such as sheet widths, frame rate, number of volumes, step-size and adaptation time were defined before starting acquisition. Volumes were acquired using z-steps of 2 μm, which was sufficient to identify ROIs corresponding to individual neuronal cell bodies (typically 5–8 μm in diameter) without an unduly high data acquisition load. Once acquired, data was reduced to time-series of fluorescence signals for each ROI correspnding to a single neuron. Operation of μSPIM Toolset was tested using more than 10 zebrafish larvae as part of another ongoing investigation.

## CRediT authorship contribution statement

**Daniel Saska:** Software, Investigation, Writing - original draft, Visualization. **Paul Pichler:** Investigation, Writing - review & editing. **Chen Qian:** Investigation. **Christopher L. Buckley:** Conceptualization, Software, Writing - review & editing, Project administration, Funding acquisition. **Leon Lagnado:** Conceptualization, Methodology, Software, Visualization, Writing - review & editing, Project administration, Funding acquisition.

## Declaration of Competing Interest

Authors have no competing interests to declare.
